# WiSE CRT Is Beneficial for Heart Failure Patients as a Rescue Therapy: Evidence From a Meta-Analysis

**DOI:** 10.3389/fcvm.2022.823797

**Published:** 2022-03-15

**Authors:** Jiehui Cang, Yaowu Liu, Didi Zhu, Shangshang Liu, Junxian Shen, Hongyu Miao, Qianxing Zhou, Long Chen

**Affiliations:** Department of Cardiology, Zhongda Hospital of Southeast University, Nanjing, China

**Keywords:** WiSE system, CRT, leadless cardiac pacing, endocardial pacing, heart failure

## Abstract

**Background:**

Leadless endocardial left ventricular (LV) pacing resynchronization therapy is a novel solution for patients with heart failure (HF) in whom conventional cardiac resynchronization therapy (CRT) failed.

**Methods:**

PubMed and the Cochrane Library were searched for relevant cohort studies. Clinical outcomes of interest such as ejection fraction (EF), QRS duration (QRSd), and left ventricular end-systolic volume (LVESV) were extracted and analyzed.

**Results:**

Five studies involving 175 HF patients for WiSE CRT were included, and patients were followed-up for 6 months. The implanted success rate ranged from 76.5 to 100%. WiSE CRT resulted in significantly narrower QRSd [mean difference (MD): −38.21 ms, 95% confidence interval (CI): −44.36 to −32.07, *p* < 0.001], improved left ventricular ejection fraction (MD: 6.07%, 95% CI: 4.43 to 7.71, I^2^ = 0%, *p* < 0.001), reduced left ventricular end-systolic volume (MD: −23.47 ml, 95% CI: −37.18 to −9.13, *p* < 0.001), and reduced left ventricular end-diastolic volume (MD: −24.02 ml, 95% CI: −37.01 to −11.03, *p* = 0.02).

**Conclusion:**

Evidence from current studies suggests that leadless endocardial LV pacing resynchronization is effective for HF patients who failed conventional CRT or needed a device upgrade, and it may be an interesting rescue therapy.

## Introduction

Cardiac resynchronization therapy (CRT) has been proven to be an effective way to improve the prognosis and mortality of patients with cardiac dyssynchronization and heart failure (HF), which can be accomplished *via* biventricular pacing. Benefits, as there have been, 30–40% of patients do not respond to this conventional CRT (1). A metaanalysis including 150 consecutive CRT papers showed that the average rate of non-responders to CRT was about 34% (2). The common causes for the failure of traditional CRT include anatomical variations and high pacing threshold (3, 4).

Wireless stimulation endocardially CRT (WiSE CRT) system (EBR Systems, Sunnyvale, CA) is a kind of novel technology that has been approved for commercial use in Europe (5). The WiSE CRT system can pace the left ventricle *via* an endocardial receiver electrode which is placed in the left ventricle (instead of implanting a lead) and is powered wirelessly by a subcutaneous ultrasound pulse generator. The transmitter placed subcutaneously sends ultrasound to an electrode in the left ventricle, which converts the ultrasound waves into an electrical stimulation potential. The transmitter is connected to the battery *via* a cable that serves as a source of energy (6). With a very short delay (3–10 ms), the transmitter can send a preprogrammed ultrasonic pulse acoustically to the electrode. The electrode converts the ultrasonic energy into electrical energy, which is used to activate the left ventricle. Stimulation can be simultaneous and biventricular due to the endocardial stimulation site. Theoretically speaking, all cardiac stimulation systems (pacemakers, defibrillators, and “leadless” pacemakers) available in the market can be coimplanted apart from subcutaneously implanted cardioverter-defibrillator (S-ICD), which currently does not allow ventricular stimulation.

The potential benefit of WiSE CRT, when compared with conventional CRT, is the ability to pace anywhere and provide targeted pacing without consideration of specific implantation site in LV, which will simplify the implant procedure and reduce the operation time (7, 8). Also, there is no need for long-time anticoagulant therapy after implantation, which may make the management of patients more convenient. Recent studies have shown that WiSE CRT could achieve narrower QRS duration (QRSd) and higher ejection fraction (EF) in those patients who failed to conventional CRT or were defined as non-responders to conventional CRT (5, 9–12). Whether WiSE CRT could help HF patients is unclear, so we conducted the meta-analysis of this novel technology to explore whether patients can benefit from WiSE CRT as a way of rescue therapy.

## Methods

### Search Strategy

We systematically searched medical databases of PubMed and Cochrane library up to October 30, 2021, without publication status restriction. The search keywords included “WiSE or leadless” or “resynchronization” without any language restriction.

### Study Selection

Two independent authors (Jiehui Cang and Yaowu Liu) filtered the studies fulfilling the following inclusion and exclusion criteria, and discrepancies were adjudicated by consensus and discussion with the third reviewer (Long Chen):

1. Trials including patients who failed conventional CRT procedure or were defined as no-responding to CRT were included or those who were previously implanted with pacemakers/ICD and met standard indications for CRT, referred to as an upgrade, were included. All patients receiving implantation of WiSE CRT were included in this meta-analysis.
2. Clinical trials including at least more than 5 patients were included. We excluded case-control studies, historically controlled studies, and crosssectional studies.
3. Outcomes of interest: ejection fraction (EF), QRS duration (QRSd), left ventricular end-systolic volume (LVESV) were included. However, in these outcomes, EF, rather than New York Heart Association (NYHA) class was compulsory.
4. Articles published as full-length articles in English were included.
5. When duplications of the data were found, the results of the most recent publications with longer follow-up durations were included in the meta-analysis.

### Data Extraction and Quality Evaluation

After the inclusion of studies, indications of interest were extracted by Jiehui Cang. The extracted data included the details of studies and patient characteristics; LVEF, LVESD, LVEDD, and QRSd at baseline and 6-month follow-up in patients receiving WiSE CRT. The quality of non-randomized controlled studies was evaluated with the Newcastle–Ottawa Scale (NOS) (13).

### Statistical Analyses

Review Manager 5.3 (The Nordic Cochrane Centre, The Cochrane Collaboration, Copenhagen, DK) was used to analyze the extracted statistics. Categorical variables were estimated and presented as the overall proportion and confidence intervals (CIs). Continuous variables were estimated by mean difference (MD) and presented as the overall average values and CI. Statistical heterogeneity was conducted by calculating I^2^, and I^2^ > 50% was considered to indicate significant heterogeneity (14). When heterogeneity was present, sensitivity and subgroup analyses were performed to explore the possible causes. Publication bias was evaluated by visual inspection of asymmetry in funnel plots (15).

## Results

### Searching Results

**Figure 1:**
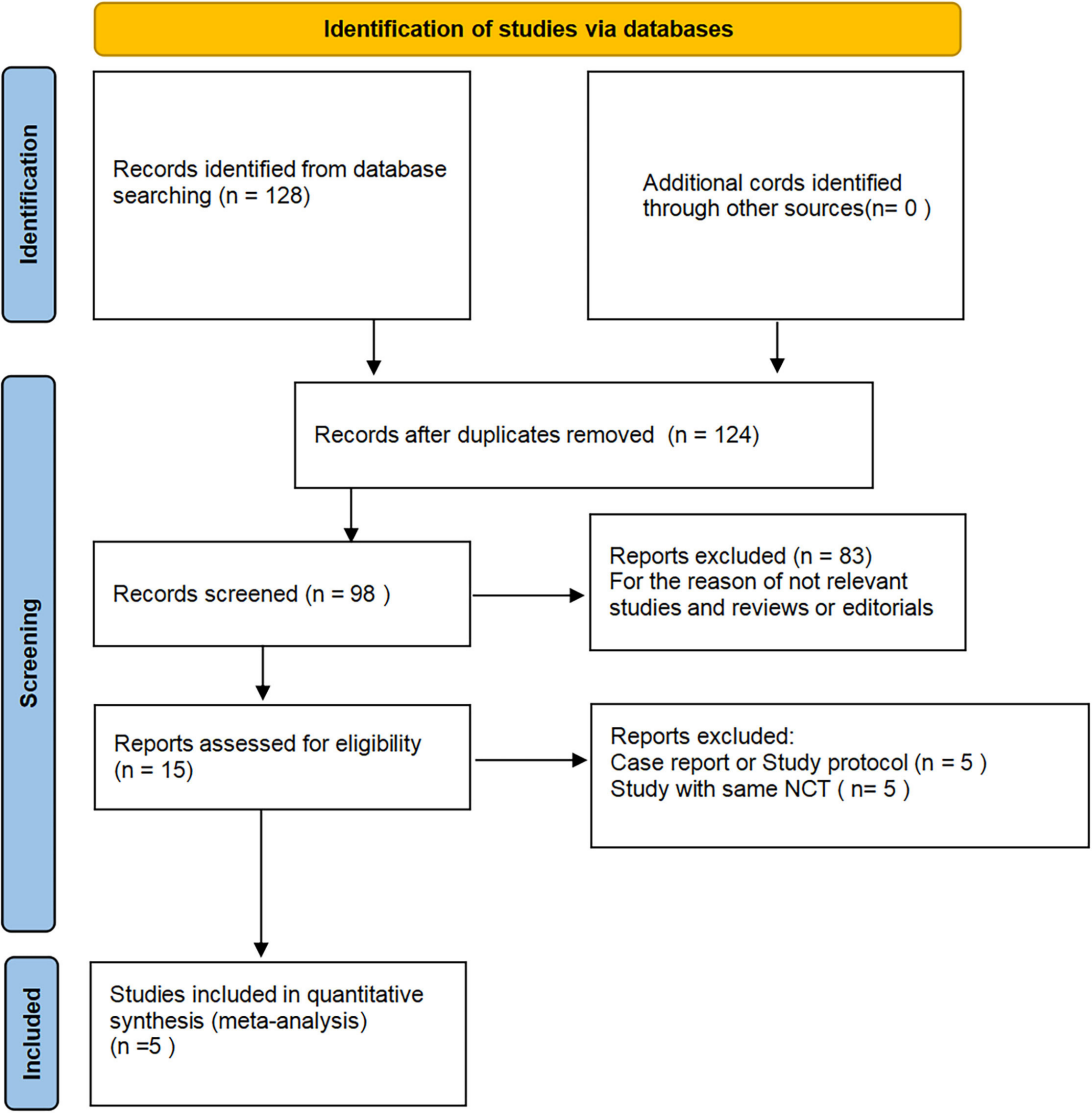
Flowchart of database search and study identification.

The process of literature searching is indicated in Figure 1. A total of 128 records were received by initial searching. A total of 15 articles were left after screening the title and abstract. The remaining records were checked by full-text review. Finally, five studies were included in the analysis (5, 9–12).

### Study Characteristics and Quality Evaluation

Overall, five single-arm studies including 175 HF patients were analyzed. All studies focusing on WiSE CRT in selected HF patients were performed between 2014 and 2021. All included patients were those who failed conventional CRT procedures or were defined as no-responding to CRT or needed a device upgrade. One retrospective study focused on the combination of Micra and WiSE CRT (11). All studies have recorded EF during follow-up. However, none of them reported procedure time or X-ray exposure time. Patients were followed for 6 months in all the included studies. The quality of five studies with the NOS ranging from 6 to 8 points, as is concluded in Table 1, was generally good.

**Table 1:** Characteristics of the included studies.

**References**	**Article type**	**Patients**	**Implant success (%)**	**NOS**	**Baseline**				**Follow-up at 6 month**			
					**QRS duration**	**LVESV (ml)**	**LVEDV (ml)**	**LVEF (%)**	**QRS duration**	**LVESV (ml)**	**LVEDV (ml)**	**LVEF (%)**
Auricchio et al. (10)	Prospective	17	76.5	6	NR	NR	NR	25 ± 0.4	NR	NR	NR	31 ± 7.0
Reddy et al. (5)	Prospective	35	97.1	8	169.9 ± 29.2	183.8 ± 62.9	243.1 ± 70.7	26.0 ± 6.2	142.6 ± 27.3	157 ± 75.7	222.4 ± 77.0	33 ± 10.3
Okabe et al. (12)	Prospective	31	100	7	177.4 ± 26.5	134.9 ± 51.3	185.4 ± 58.8	28.3 ± 6.7	152.3 ± 30.5	111.1 ± 40.3	164.9 ± 50.6	33.5 ± 6.9
Sidhu et al. (11)	Prospective	104	97.1	8	181.1 ± 28.1	131.7 ± 68.1	187.1 ± 84.8	30.8 ± 7.9	137.4 ± 28.2	108.8 ± 65.3	164.1 ± 75.3	36.7 ± 10.4
Carabelli et al. (9)	Retrospective	8	100	6	204.37 ± 30.26	117.33 ± 35.61	160.0 ± 22.69	28.43 ± 8.01	137.5 ± 24.75	91.86 ± 48.43	129.4 ± 40.7	39.71 ± 11.89

*LVEF, left ventricular ejection fraction; LVEDV, left ventricular end-diastolic volume; QRSd, QRS-wave duration; NR, not reported; LVESV, left ventricular end-systolic volume*.

### Electrophysiology Assessment

**Figure 2:**
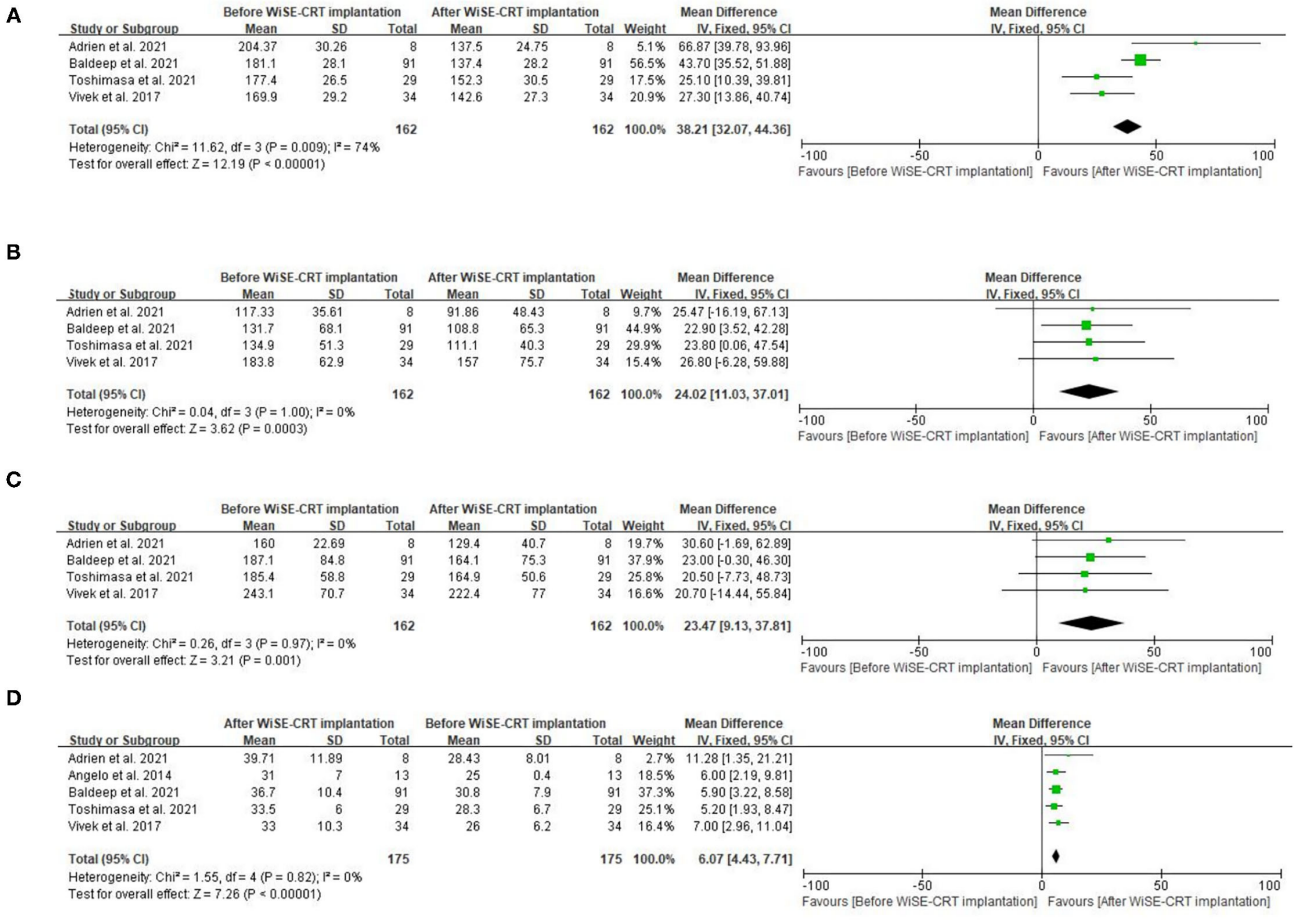
Forest plots for the meta-analysis. **(A)** QRSd, **(B)** LVEDV, **(C)** LVESV, and **(D)** LVEF.

Four studies recorded QRSd at both baseline and follow-up (Figure 2A) (5, 9, 11, 12). The average QRSd decreased significantly (MD = −38.21 ms, 95% CI: −44.36 to −32.07, *p* < 0.001) after implantation of WiSE CRT. The heterogeneity among studies was high (I^2^ = 74%). Neither subgroup analysis nor sensitivity analysis could not significantly reduce the heterogeneity, and hence we decided to exclude none of them. One study additionally showed high R-wave amplitude (5.6 ± 3.2 mV) and low electrical pacing threshold (1.6 ± 1.0 V), respectively (10).

### Echocardiography Assessment

All five studies included the assessment of LVEF after implantation of WiSE CRT, which indicated that WiSE CRT could improve cardiac function of selected patients with HF (MD = 6.07%, 95% CI: 4.43–7.71, I^2^ = 0%, *P* < 0.001) within half-one-year follow-up (Figure 2D). Echocardiography outcomes representing alterations in structure, in addition, were also focused on. Reduced LVEDV (MD = −24.02 ml, 95% CI: −37.01 to −11.03, *P* = 0.02) (Figure 2B) and reduced LVESV (MD = −23.47 ml, 95% CI: −37.18 to −9.13, *P* < 0.001) (Figure 2C) were displayed at the 6-month follow-up, both of which were improved prominently when compared with baseline. Moreover, the fixed-model was applied for the evaluation of echocardiographic index, whereas no heterogeneity was observed (I^2^ = 0).

### NYHA Class Assessment

Three studies indicated the individual change of NYHA class. One study showed that NYHA class of 50% patients were moderately or markedly improved, while another study presented that 69.7% of patients achieved great or moderate improvement (5, 10). The third study only showed that ≥1 NYHA class improvement was accomplished in 46.7% of patients (12). Two studies gave the result of comparison of NYHA class at follow-up vs. that at baseline, and there seemed to be a conflict between the two studies. Sidhu et al. found a significant reduction in NYHA functional class (2.6 ± 0.5 vs. 2.1 ± 0.6; *P* < 0.001), while Carabelli et al. reported that NYHA functional class was not well improved (2.63 ± 0.51 vs. 2.29 ± 0.95; *P* = 0.18) (9, 11).

### Safety Outcomes

A total of three studies reported complications, which included 53 device- or procedure-related adverse events after implantation (5, 10, 12). Two patient deaths due to peri-procedural pericardial effusion and ventricular fibrillation (VF) during the procedure were reported by Auricchio et al. and Reddy et al., respectively. During the 6-month follow-up, 5 patients with defective transmitter circuitry were reported. No thrombo-embolic strokes occurred, except in one patient with atrial fibrillation (AF) who did not accept anticoagulant therapy. In addition, no lead dislodgements, loss of capture, ventricular septal perforation, device-related infection, or coronary artery injury were observed.

### Publication Bias and Sensitivity Analysis

**Figure 3:**
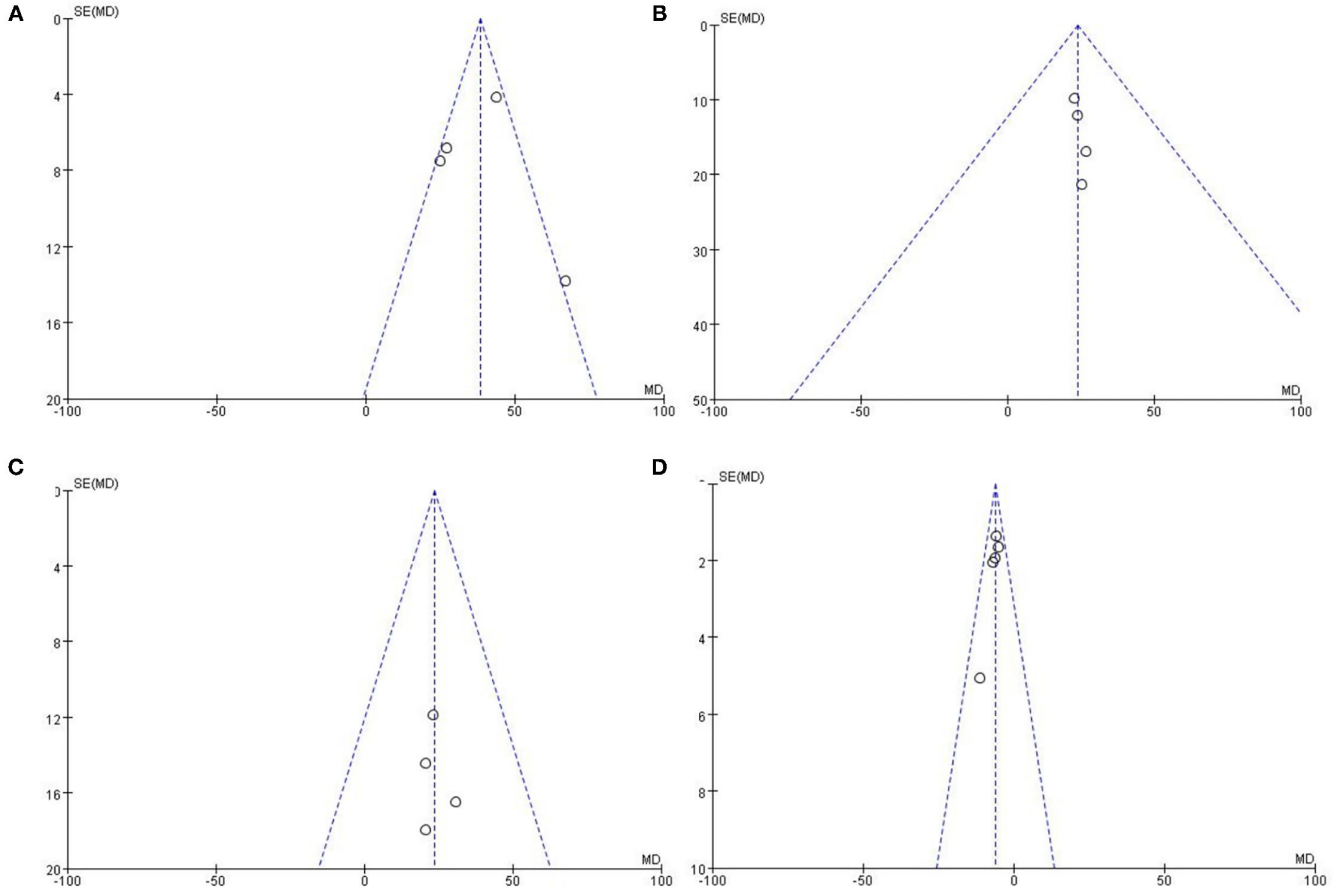
Funnel plots for the meta-analysis. **(A)** QRSd, **(B)** LVEDV, **(C)** LVESV, and **(D)** LVEF.

Funnel plot (Figure 3) of the included studies indicated no obvious publication bias. Sensitivity analyses were accomplished by systematically excluding one study at a time, and no single study affected most of the above results.

## Discussion

This meta-analysis, by pooling five relevant researches evaluating 175 HF patients, assessed current publications on the efficacy and safety of WiSE CRT in selected patients who failed in conventional CRT or needed a device upgrade. To the best of our knowledge, this study is the first meta-analysis to evaluate the feasibility of WiSE CRT in selected patients, and our results indicated that: (1) WiSE CRT, to some extent, was practical for patients who failed conventional CRT or for those who needed an upgrade; (2) WiSE CRT could conduce to narrow QRSd; (3) WiSE CRT could improve LVEF to a marked extent and be of great help to inhibit the remodeling of heart; (4) WiSE CRT could effectively reduce symptoms of HF patients, which was judged *via* NYHA class. Considering the above findings, WiSE CRT appears to be a good option for HF patients who fail the conventional CRT or need an upgrade.

WiSE CRT was firstly reported by Auricchio et al., which was a rescue therapy for three patients: (i) a patient needing an upgrade; (ii) a CRT patient whose LV lead does not capture, and (iii) a CRT patient classified as a non-responder, respectively (16). In that case, all three patients were successfully treated with a great improvement in LVEF (from 23.7 ± 3.4% to 39 ± 6.2%; *P* < 0.017) at the 6-month follow-up. Subsequently, Auricchio et al. carried on the first multicener, prospective study on the feasibility, safety, and short-term outcome of WiSE CRT (10). A total of 17 patients were included and 13 patients were finally analyzed. This study showed that WiSE CRT delivered a great increase in LVEF (from 25 ± 4.0% to 31 ± 7.0%, *P* < 0.01) during a 6-month follow-up. Another study carried out by Baldeep et al. tried to indirectly compare WiSE CRT implantation with coronary sinus (CS) upgrades achieved by epicardial LV lead placement in the CS (11). The study showed that CS upgrades were more likely to have an absolute change in LVEF with less burden of comorbidities. However, the study was not powerful enough to prove whether WiSE CRT was more excellent than CS upgrade or not since it did not compare CS upgrade with WiSE CRT directly. Considering the aims of our studies, we finally decided to include one retrospective study after carefully reviewing and evaluating the quality of it (11). Improvement of chamber volume and EF observed in our meta-analysis indicated that WiSE CRT might be an effective strategy for resynchronization therapy and it can be a good rescue therapy for patients with HF who failed traditional CRT or needed an upgrade. The SOLVE-CRT (Stimulation Of the Left Ventricular Endocardium for Cardiac Resynchronization Therapy, NCT02922036), which contained 350 HF patients is an international, multicenter, randomized, double-blind, sham-controlled trial of patients with Class I and IIa indications for CRT who have either failed to respond to or have been unable to receive conventional CRT, and therapeutic effects of WiSE CRT on LVEF and clinical outcomes may have been recently published (17).

However, two deaths were observed in these studies. One patient died as a result of long-time resuscitation, and another one resulted from pericardial effusion, which might be related to the process of procedures. During the initial implantation in patients, there was a risk of pericardial tamponade due to myocardial injury, and hence patients should be selected carefully before implantation and those with high risks such as myocarditis should be evaluated carefully. Also, 5 patients with defective transmitter circuitry were reported, which indicates that further development of the device is needed. Additionally, more than 50 adverse events were observed in these studies. These problems could be eliminated by the further development of the implantation procedure.

WiSE CRT is an innovative technology to achieve endocardial LV resynchronization wirelessly by a subcutaneous ultrasound pulse generator. A new technical approach called ultrasound-mediated stimulation is applied, while the mean ultrasound-mediated pacing threshold mechanical index it utilizes was only 0.5, which is far away from the index needed for imaging (18). Due to the endocardial stimulation site, stimulation can be simultaneous and biventricular, which is the reason why WiSE CRT can achieve narrower QRSd. On the one hand, the advantages of WiSE CRT lie in a very small, completely endothelialized left ventricular electrode, which itself does not require a battery, and other components of the system are extravascular, so they are easy to be removed in the event of infection and without higher risks. On the other hand, WiSE CRT system provides LV endocardial resynchronization pacing without the need for permanent oral anticoagulation. Moreover, WiSE CRT, unlike traditional pacemakers, is not vulnerable to interference from the use of electromagnetic energy sources due to ultrasound transmission (19). WiSE CRT system is therefore a potentially interesting device for the group of CRT upgrade candidates. It is also an interesting rescue therapy for those so-called non-responders or patients who have failed conventional CRT. So far, all researches related to WiSE CRT have paid attention to “non-responder” and patients who need an upgrade, and no trials directly compare WiSE CRT with conventional CRT, so it remains to be seen which patients are the most interesting population for WiSE CRT, and whether this new technology will be superior to conventional CRT or not. Hence, more related clinical trials are needed to explore the application conditions of WiSE CRT.

His-bundle pacing (HBP) was firstly reported by Deshmukh et al. (20). HBP has emerged as an ideal form of physiological pacing as it activates the normal cardiac conduction system resulting in synchronized contraction of ventricle. Left bundle branch pacing (LBBP), as an alternative way to overcome the limitations of HBP such as lead stability, higher threshold, and early battery depletion, provides low and stable pacing threshold, lead stability, and correction of distal conduction system disease (21). Both of them are more and more popular due to their ability to accomplish physiological pacing. However, the procedural process of HBP and LBBP are complex, and comorbidities such as septum perforation, conduction system injury, and septal artery injury need to be addressed. To some extent, WiSE CRT system can finish simultaneously pacing without damage to the septum, which enables it to avoid those comorbidities theoretically. However, whether WiSE CRT is superior to HBP or LBBP is not clear, and no relevant research has been found so far. So more clinical trials which compare WiSE CRT with HBP or LBBP are needed to explore the superiority of WiSE CRT.

Feasibility, as it has been shown in resynchronization therapy, WiSE CRT has its limitations. Firstly, ultrasound intensities may be decreased by attenuation, refraction, or reflection of acoustic energy from the rib or lung during the procedure due to inappropriate use of transthoracic echocardiography, which will ultimately have a bad effect on the longevity of the battery (16). Though the battery life has been extended to about 4 years so far, there is an urgent need to make this system more efficient and to develop its battery further. Secondly, WiSE-CRT system requires 2 chest wall incisions and retrograde arterial access, which will predispose to infectious complications. Thirdly, whether the WiSE-CRT system can achieve left bundle branch pacing and the efficacy of left bundle branch pacing operated by it is still unknown, even though endocardial pacing may potentially be more beneficial due to site-specific pacing theoretically. Fourthly, all studies related to WiSE-CRT selected patients who were no-responding to CRT or needed an upgrade, and no trials have directly compared WiSE-CRT with conventional CRT. So whether WiSE-CRT can be superior to conventional CRT is still unclear. Last but not least, injections to left ventricles such as left ventricle perforation and pericardial effusion would occur if the sheath is manipulated inappropriately during the anchor of the electrode, especially in those patients with diffuse myocardial disease (16, 22).

## Limitations

Our meta-analysis has some limitations. Firstly, the number of included patients was quite limited, and the included studies were non-randomized trials, which may reduce the power to validate our findings. Secondly, patients included in the studies were all followed up for 6 months, and so the potential clinical benefits from a longer follow-up duration is unknown. Besides, as we mentioned before, heterogeneity was found when we analyzed QRSd which might result from the measurement of QRSd.

## Conclusion

Results of this meta-analysis of current studies suggest that leadless endocardial LV pacing resynchronization appears to be a promising method of rescue therapy for HF patients who failed to conventional CRT or needed a device upgrade. WiSE CRT is of great possibility to achieve narrow QRSd and to improve remodeling of the heart. However, WiSE CRT should be developed further to extend its application. Also, more related clinical trials are needed to explore the most interesting population for WiSE CRT and whether WiSE CRT is superior to conventional CRT or physiological pacing such as LBBP or HBP.

## Author Contributions

JC designed the study. JC and YL performed the database search, study identification, quality evaluation, data extraction, and drafted the manuscript. JC and SL performed the statistical analyses. All authors interpreted the results, revised, and approved the submission of the manuscript.

## Conflict of Interest

The authors declare that the research was conducted in the absence of any commercial or financial relationships that could be construed as a potential conflict of interest.

## Publisher's Note

All claims expressed in this article are solely those of the authors and do not necessarily represent those of their affiliated organizations, or those of the publisher, the editors and the reviewers. Any product that may be evaluated in this article, or claim that may be made by its manufacturer, is not guaranteed or endorsed by the publisher.
